# Perspective on Theoretical Modeling of Soft Molecular Machines and Devices: A Fusion of Data‐Driven Approaches with Traditional Computational Chemistry Algorithms

**DOI:** 10.1002/open.202500229

**Published:** 2025-07-09

**Authors:** Costantino Zazza, Nico Sanna, Stefano Borocci, Felice Grandinetti

**Affiliations:** ^1^ Department for Innovation in Biological, Agro‐food and Forest systems Università della Tuscia (DIBAF) L.go dell’Università, s.n.c. 01100 Viterbo Italy; ^2^ Istituto per la Scienza e Tecnologia dei Plasmi del CNR (ISTP) Via G. Amendola 122/D 70126 Bari Italy; ^3^ Istituto per i Sistemi Biologici del CNR (ISB), Sede di Roma ‐ Meccanismi di Reazione c/o Dipartimento di Chimica Sapienza Università di Roma P.le A. Moro 5 Rome Italy

**Keywords:** data‐driven approaches, density functional theories, molecular machines and devices, quantum mechanical/molecular mechanical methodologies, quantum theory of atoms in molecules

## Abstract

The design of complex molecular machines and devices represents one of the most ambitious frontiers in nanotechnology, synthetic chemistry, and molecular engineering. These intricate systems, inspired by biological machines, require precise control over atomic and electronic interactions to achieve desired functionalities. Theoretical modeling plays a crucial role in this process, offering predictive insights into molecular behavior, guiding experimental design, and optimizing performance. Methods such as density functional theory, quantum theory of atoms in molecules coupled with widely adopted and distinctive visualization methods, molecular dynamics simulations, and quantum mechanical/molecular mechanical hybrid approaches provide analytical information into the stability in terms of mutual chemical interactions and conformational shaping of flexible supramolecular aggregates for nanotechnological applications. Theoretical approaches also facilitate interdisciplinary integration, bridging chemistry, physics, and materials science to create conceptually hybrid devices with enhanced performance. Machine learning and artificial intelligence are now being incorporated into theoretical modeling, accelerating the discovery and refinement of novel molecular architectures. This fusion of data‐driven approaches with traditional computational chemistry algorithms is expected to revolutionize the design paradigm of soft molecular machines and devices.

## Graphical abstract

Theoretical approaches facilitate interdisciplinary integration, bridging chemistry, physics, and materials science to create conceptually hybrid devices with enhanced performance. Machine learning and artificial intelligence are now being incorporated into theoretical modeling, accelerating the discovery of novel molecular architectures. This fusion is expected to revolutionize the design paradigm of soft molecular machines and devices.

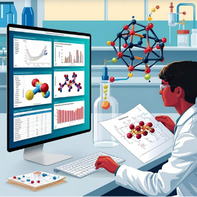

## 1 Introduction

The design and optimization of molecular devices and machines are among the most exciting frontiers in modern science and technology, with applications ranging from molecular electronics and sensors to molecular motors.^[^ ^1^, ^2^, ^3^ ^]^ The development of these systems requires a deep understanding of how individual molecular components interact, how energy is transferred between them, and how external stimuli influence their behavior in different contexts.^[^ ^4^, ^5^, ^6^, ^7^, ^8^ ^]^ However, due to the complexity of molecular interactions finely modulating the conformational shaping and the associated stimuli‐responsive properties, experimental techniques albeit indispensable nowadays benefit from self‐consistent theoretical methods that results useful for a rational design of molecular machines of increased efficiency and desired functionalities.^[^ ^9^, ^10^, ^11^ ^]^ Density functional theory (DFT) has emerged as one of the most powerful and widely used methods for simulating molecules with a few hundred of atoms.^[^ ^12^, ^13^, ^14^ ^]^ DFT therefore plays an active role in the design of the electronic properties of molecular machines, offering a good compromise between efficiency and accuracy. Its ability to predict some key chemical‐physics properties—such as electronic energy levels, charge distribution, and reactivity—makes it an indispensable tool for understanding and making a prediction about the performance of molecule‐size devices even in the presence of different substituents. Another key factor that can influence the effective operation and usage of molecular machines and devices is the solvent.^[^ ^1^, ^2^, ^3^, ^6^, ^11^ ^]^ Mutual interactions with an encircling solvent can lead to profound changes in their functionality, often dictating how efficiently they work and how adaptable they are to different environments. The effects of solvents are multifaceted, affecting properties such as molecular recognition, charge transfer process, conformational shaping, and overall stability.^[^ ^15^, ^16^, ^17^, ^18^, ^19^ ^]^ Solvent effects can even impact their solubility and self‐assembly behavior.^[^ ^1^, ^2^, ^3^, ^20^, ^21^, ^22^, ^23^ ^]^ Solvent molecules can act as mediators for energy transfer or catalysis, facilitating specific molecular interactions that are required for the proper function of the machine itself.^[^ ^6^, ^24^, ^25^, ^26^ ^]^ One of the primary advantages of DFT when combined with both implicit and explicit solvation effects is its ability to handle large systems with a reasonable computational cost. A critical feature of DFT is its use of the electron density as the central variable, rather than the many‐body wave function used in other quantum mechanical (QM) methods. Various functionals—mathematical approximations of the exchange‐correlation potential—are employed to describe the interactions between electrons, and these functionals can be tailored to the specific needs.^[^ ^12^, ^13^, ^14^ ^]^ Another key benefit of DFT is its ability to model dynamic processes. In molecular machines, for example, the motion of components is often critical to the overall system's function. Such a technique can be used in combination with molecular dynamics propagators^[^ ^27^ ^]^ to provide insights into the energetic barriers between transient states, helping researchers identify the most efficient pathways for motion and energy transfer.^[^ ^15^, ^28^, ^29^, ^30^, ^31^ ^]^

Molecular machines are often designed to work in concert with other components, and DFT can be used to explore how these components interact with one another with the support of the quantum theory of atoms in molecules (QTAIM) technique.^[^ ^32^, ^33^, ^34^ ^]^ This method enables the identification of critical points, such as bond paths and electron density maxima, which are essential for understanding peculiar stability patterns in supramolecular aggregates of interest. In this respect, QTAIM has been recently applied for an analytical characterization of chemical contacts modulating the conformational changes observed in H‐shaped molecular shuttles in the presence of metal cations;^[^ ^35^ ^]^ visual analysis methods of covalent and noncovalent interactions in real space—such as the noncovalent interaction decomposition, the interaction region indicator, the independent gradient model with its variants are also becoming popular due to their intuitive pictorial view of chemical interactions even in the presence of complex molecular systems.^[^ ^36^, ^37^ ^]^ QM/ molecular mechanical (MM) methodologies are also emerging as self‐consistent tools for addressing spectroscopic properties of soft molecular machines in different environments.^[^ ^38^, ^39^, ^40^, ^41^, ^42^, ^43^ ^]^ These methodologies are hybrid techniques that partition a molecular system into two distinct regions: a QM‐treated region, where electronic structure calculations are performed with high accuracy, and an MM‐treated region, where classical force fields are applied to simulate the larger molecular environment. This dual approach is seen to provide a balanced compromise between computational efficiency and chemical accuracy, making it particularly useful for studying flexible molecular machines with complex electronic and spectroscopic behaviors.^[^ ^44^ ^]^ In this context, recent developments in QM/MM algorithms including sophisticated coupling schemes between QM and MM regions have led to more realistic simulations, accounting for polarization effects and dynamic correlations within complex molecular environments. Moreover, the development of adaptive QM/MM schemes, where the QM region dynamically adjusts based on system requirements, represents another promising direction.^[^ ^41^, ^45^, ^46^, ^47^ ^]^

**Figure 1:**
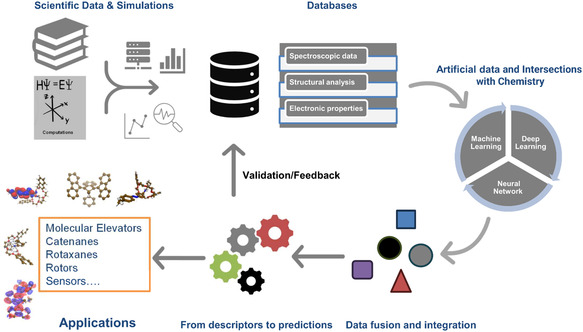
Schematic view of the hypothesized workflow combining scientific literature and traditional computational methodologies with data‐driven algorithms for nanomaterials discovery. Traditional computations thus serve as self‐consistent tools to generate data and information across many length and time scales (from QM to MM approximations). Robust AI models are then constructed from comprehensive and structured datasets to approximate underlying high‐dimensional descriptors intrinsic to chemical or supramolecular properties. This process actually allows to identify compounds with desired properties when subject to certain external stimuli (e.g., a specific thermodynamics stability and/or solubility, an optical spacing for efficiently absorbing photons within an imposed energy range, a particular conformational shaping under exercise conditions etc.). In turn, molecular systems with potential nanotechnological applications will be selected for subsequent studies and validations in the laboratory.

Machine learning has shown promise in accelerating the search for potentially relevant molecular systems for nanotech applications by training models based on previously calculated data, typically at QM‐level of accuracy.^[^ ^48^, ^49^, ^50^, ^51^ ^]^ These models can efficiently analyze vast chemical databases (if available) to discern patterns governing molecular behavior, thus enabling the rapid generation of novel molecular candidates with desired characteristics. Moreover, generative models such as variational autoencoders^[^ ^52^, ^53^ ^]^ and generative adversarial networks^[^ ^54^, ^55^, ^56^, ^57^ ^]^ are expected to facilitate the discovery of innovative molecular architectures that would be challenging to conceive through conventional theoretical methods. In addition, Deep learning architectures such as graph neural networks might also be useful, as they model molecular structures as graphs where atoms act as nodes and bonds as edges.^[^ ^58^, ^59^ ^]^ These structured networks can effectively capture complex molecular features and interactions, enabling researchers to simulate reaction pathways, assess supramolecular assembly, and optimize suggested configurations. Despite the transformative potential of AI, challenges such as data limitations, model interpretability, and generalization still remain an open question. Ensuring high‐quality datasets, enhancing explainability in AI models, and integrating AI with more traditional computational algorithms are crucial steps for future advancements (see **Figure** **1**).^[^ ^60^, ^61^, ^62^, ^63^ ^]^ AI‐quantum models capable of providing accurate approximations of electronic structure wave functions and underlying potential energy landscapes can in fact make simulations accessible that would otherwise be very challenging, thus facilitating studies on molecular systems of gradually increasing complexity contributing to the development of next‐generation molecular machines. Beyond computational design, automated synthesis powered by AI‐driven retrosynthetic analysis will make it possible to identify optimal reaction pathways predicting feasible synthetic routes based on existing reaction databases; this will drastically reduce the time and resource consumption in the laboratory during the formulation of synthetic prototypes. The interdisciplinary nature of AI‐driven molecular design also necessitates collaboration and data exchange between computational scientists, chemists, material scientists, and engineers with the target of introducing a paradigm shift in scientific research and correlated developments. By enabling efficient molecular modeling, predictive analysis and synthesis automation, AI could conceivably speed up the identification of complex systems with potential nanotechnological implications with unprecedented precision. By addressing existing challenges and leveraging AI's full potential, researchers can unlock new frontiers in molecular technology, establishing a new era of intelligent molecular design that will redefine scientific and technological possibilities in the intriguing field of stimuli‐responsive molecular devices. A fruitful fusion of established, or traditional, methodologies with emerging data‐driven ones can undoubtedly open up new perspectives in the modeling of advanced nanoscale machines according to the opportunities that will arise in the near future. As a result, we expect that interoperability between traditional and emerging technologies may eventually facilitate the release of more environmentally friendly molecular machines by reducing unfeasible laboratory activities.

## Conflict of Interest

The authors declare no conflict of interest.
